# Investigation of physicochemical characteristics of selected lignocellulose biomass

**DOI:** 10.1038/s41598-022-07061-2

**Published:** 2022-02-21

**Authors:** M. O. Fajobi, O. A. Lasode, A. A. Adeleke, P. P. Ikubanni, A. O. Balogun

**Affiliations:** 1 Department of Mechanical Engineering, Faculty of Engineering and Technology, Ladoke Akintola University of Technology, Ogbomoso, Nigeria; 2 Department of Mechanical Engineering, Faculty of Engineering and Technology, University of Ilorin, Ilorin, Nigeria; 3 Department of Mechanical Engineering, College of Engineering, Landmark University, Omu-Aran, Nigeria

**Keywords:** Biotechnology, Mechanical engineering

## Abstract

The beneficial effects of biofuels as components of the worldwide energy supply are unquantifiable because they have versatile applications. However, an adequate understanding of the chemical properties of typical biomass is an integral aspect of maximizing the energy potentials because it is susceptible to biomass behavior during the conversion process, especially anaerobic digestion. Therefore, this study investigated the physicochemical characteristics of selected lignocellulose biomass, namely; cow dung, mango pulp, and *Chromolaena odorata* of Nigerian origin. The raw biomasses were characterized by proximate, calorific, ultimate, compositional, and microbial (for cow dung only) analyses using ASTM standards and equipment. Raw biomass characterization showed that cow dung, mango pulp, and *Chromolaena odorata leaves* recorded percentages; fixed carbon, volatile matter, and ash contents in addition to calorific values in the ranges of 6.22–7.25%, 5.02–7.79%, 1.14–1.91,% and 13.77–16.16 MJ/kg, respectively. The ultimate analysis of cow dung, mango pulp and Chromolaena odorata recorded carbon (43.08, 39.98, 41.69%); hydrogen (7.87, 6.74, 9.86%); nitrogen (1.53, 1.34, 1.51%); sulphur (0.46, 0.12, 0.25%) and oxygen (47.06, 51.82, 46.69%), respectively. Compositional analysis of the biomass gave percentages in the range of 7.47–11.37 for hemicellulose, 0.22–6.33 for lignin, and 3.71–12.03 for cellulose, while the microbial analysis of cow dung gave total bacteria counts of 5.78 × 10^8^ and 3.93 × 10^5^ cfu/g on wet and dry bases, respectively, which implied that it was rich in microbial colonies, evidently from the various species found, such as *Escherichia coli, Staphylococcus aureus, Bacillus cereus, Pseudomonas aureginosa, Proteus morganii, and Micrococcus *spp. In this regard, the physicochemical properties of selected biomass of Nigerian origin were established to conform with those of the literature and thus can be regarded as suitable feedstock for anaerobic digestion resulting in methane-rich biogas products.

## Introduction

The world population was estimated to be 7.6 billion in 2016 and is projected to grow at an average rate of 1.09% yearly^1^. Annually, the global energy consumption on the average increases by 2%, leading to a twofold increase in energy consumption every 35 years^2^. This demonstrates a directly proportional relationship between the world growth rate and the global energy demand. Over 80% of the energy being presently utilized globally emanates from conventional energy sources such as fossil fuels^3^; however, the combustion of these fuels generates hazardous products that lead to adverse environmental effects. To tackle this problem, many researchers have the quest for alternative energy sources (especially those of biomass-origin) that are environmentally compatible, suitable for sustainable development, and can augment the present energy mix. According to Omoniyi and Olorunnisola^4^, biomass is defined as organic materials mostly considered to be wastes since they do not directly go into foods or consumer products. Biomass includes kitchen residues, sewage sludge, farm residues, disposed environmental refuses, livestock droppings, organic fraction of municipal solid residue, vegetable market waste, agricultural and food wastes, animal manure, cow dung, mango pulp, green leaves, rice husks, pawpaw peels, and potatoes peels^5–8^. Biomass is composed of high volatile matter, which can be converted to other useful gaseous and liquid biofuels through thermochemical processes such as pyrolysis, bio-gasification, and combustion^9^. The versatility of biomass is acknowledged across the world but is inadequately managed, thus causing nuisance and other menaces to host communities. Therefore, due to the degree of such biomass availability and their potentials, large-scale energy recovery from biomass with no perceived consequences on both human activity and the environment is strongly encouraged^10^. Biomass is not only of interest as an energy source but is steadily turning to an alternative raw material used for power and biobased products such as building materials and chemicals and in the mass production of plastics^11^. They are also advantageous because they are renewable energy sources that are not associated with environmental hazards such as radioactive waste disposal, open pits, acid rain, or mine spoils^4^. Efficacies of diverse biomasses and their sustainability and suitability have been well established by many researchers. For instance, in a study by Demirbas^12^, who investigated the comparison of mixtures of manure and straw to manure alone as biomass for bio gasification at mesophilic temperatures. The physicochemical characteristic of the biomass in terms of percentage methane contents were reported to be 14.7% and 10.4%, respectively. The energy content of approximately 33–50% of the higher calorific value was recorded. Another study conducted by Kobra et al*.*^13^ employed an adaptive neuro-fuzzy for modeling experimental data generated from laboratory biogas production using kitchen wastes. The predictive model developed was validated and found to accurately predict the experimental data, evidently from the obtained values for both the coefficient of determination, R^2^, and adjusted coefficient of correlation, with adjusted R^2^ values of 0.9946 and 0.9927, respectively. Additionally, Wannapokin et al.^14^ used fallen teak leaves and *Tectona grandis* to generate biogas*.* It was reported that leaves are suitable biomass capable of generating biogas, particularly when adequately digested anaerobically. The ultimate analysis of the fallen teak leaves gave C, 48.88%, H, 5.83%, N, 0.55%, S, 0.18%, and O, 30.04%. On a dry basis, proximate analysis recorded a moisture percentage of 2.83%, ash content of 11.33%, volatile matter content of 83.44%, and fixed carbon content of 2.4%. Optimum biogas compositions were established and reported to be 43.57 and 55.47% carbon dioxide and methane, respectively. Generally, indigenous biomass characterization is of paramount importance because of peculiarities of locations, atmospheric conditions, and nutrition because the bioefficacy of any selected biomass for energy extraction is informed by these factors. Lignocellulose biomass as reported by Uzodinma et al.^15^ and Agus et al.^16^, can be blended to have a significant improvement on biogas yield both quantitatively and qualitatively through a synergistic effect. Research findings established that biomass blends such as kitchen refuse and domestic sewage^17^, brewery spent grain and poultry droppings^18^, brewery spent grain, carbonated soft drink, powdered rice husk and soya bean cake^15^, Ilama, sheep and cow manure^19^, cow dung and rice husk^20^, cattle excreta and two-phase olive mill wastes^21^, crude glycerine obtained from biodiesel and cattle dung^22^, horse and cow dung^23^, maize leaves and elephant grass^24^, cattle dung with plantain peels^25^, *Justicia schimperiana* and cow dung^26^, cow dung and elephant grass^15^, amongst others yielded improved results compared to sole digestion of each of the biomass investigated. Apart from fortification of required elemental composition through blending because biomass complements themselves in terms of characteristics, the stability of the digestion process, is also enhanced through biomass blends. This, suggests the need for adequate characterization of selected biomass to establish their suitability as feedstock for anaerobic digestion resulting in improved biogas production. Therefore, with such diversified biomass sources, energy production techniques, and sketchy information on the suitability of cow dung, mango pulp, and *Chromolaena odorata* as anaerobic digestion feedstocks, it becomes imperative to understand their physicochemical characteristics through standard procedures, hence the conception of this study. This study predominantly aimed to characterize the highlighted biomass using proximate, ultimate, compositional, calorific value, and microbiological content analyses. The results of this study will serve as a guide for biogas producers and allied who wish to extract energy from the investigated biomass.

## Materials and methods

### Biomass collection

**Figure 1:**
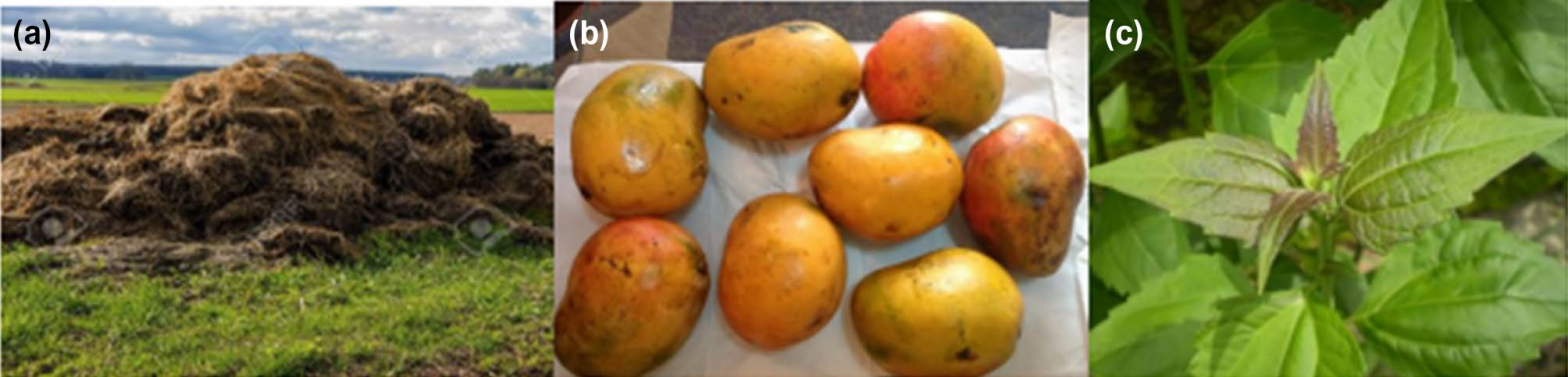
Raw biomass (**a**) cow dung (**b**) mangoes and (**c**) *Chromolaena odorata leaves*.

Three major biomasses were characterized in this study: cow dung, waste mango (Cherry species), and *Chromolaena odorata* leave (Fig. 1). Fresh cow dung was collected from a prominent abattoir named *Atenda.* Mango was obtained from the *Kinnira* area, while *Chromolaena odorata* leaves (locally known in the area as *Ewe Akintola*) were collected at the Ikose area. All collection points are located at Ogbomoso (8° 8′ 31.79″ N, 4° 1′ 42.67″ E), southwestern Nigeria.

### Proximate analyses of biomass

Proximate analysis was conducted on biomass materials to determine the moisture content (MC) ash content (AC), volatile matter (VM), and fixed carbon (FC). Proximate analysis of the selected biomass was carried out at the Institute of Agricultural Research and Training, IAR&T, Ibadan, Nigeria. All analyses were carried out in triplicate, and the average values were reported.

### Determination of moisture content

The moisture contents of the selected biomass were determined using ASTM D4442-16^27^ standard. A 2 g quantity of biomass sample was initially weighed before being poured into a preweighed ceramic crucible (Model No: ${Al}_{2}{O}_{3}$, Standard Advanced Materials, USA). Subsequently, the crucible containing the biomass sample was weighed. The samples were dried at 105 °C in an oven for 2 h. Then, the samples were subjected to cooling in a standard desiccator (Model No: DSGL150) and later weighed while the moisture loss was documented. Equation (1) was used to determine the moisture contents in the biomass.

1 $$\mathrm{MC}= \frac{{\mathrm{W}}_{2}- {\mathrm{W}}_{3}}{{\mathrm{W}}_{2}- {\mathrm{W}}_{1}} \times 100 {\%}$$

where ${W}_{1}$ = Empty crucible weight (Initial or previous), ${W}_{2}$ = Crucible weight and biomass sample before oven drying, and ${W}_{3}$ = Crucible weight and biomass sample afterwards oven-dried.

### Determination of ash content

The quantity of solid residue after the biomass sample is completely burnt represents the ash content. The ASTM E1755-01^28^ standard was adopted to determine the ash content in the selected biomass. A 2 g sample was decanted into a preweighed standard crucible and then combusted (incinerated) in a muffle furnace (Model No: OF-22G, JESO TECH, Korea) at 760 °C until complete ash content or white greyish matter was attained. The crucible was then allowed to cool in a standard desiccator (Model No: DSGL150) and reweighed. The ash content was determined using Eq. (2).

2 $$Ash \; Content= \frac{{W}_{3}- {W}_{1}}{{W}_{2}- {W}_{1}} \times 100\%$$

where ${W}_{1}$ = previous weight of empty crucible, ${W}_{2}$ = crucible weight, and biomass sample before combustion (incineration), and ${W}_{3}$ = crucible weight and biomass sample after combustion (incineration).

### Determination of volatile matter

When the biomass is heated, the condensable vapour and permanent gases (exclusive of water vapour) released as a result of heating are known as volatile matter. The ASTM E872-82^29^ standards were followed to obtain the biomass volatile matter. A 2 g sample of each biomass was poured into a standard crucible and heated in a muffle furnace (Model No: OF-22G, JESO TECH, Korea) at 800 °C for 7 min. It was then cooled in a standard desiccator (Model No: DSGL150) and reweighed. The weight loss due to devolatilization is regarded as the volatile matter which was determined using n Eq. (3).

3 $$Volatile \,Matter\,Content = \frac{{W}_{1} - {W}_{2} }{{W}_{1}} \times 100\%$$

where ${W}_{1}$ = Previous weight of the sample and ${W}_{2}$ = Sample final weight after incineration.

### Determination of fixed carbon

The fixed carbon content for the biomass was calculated using Eq. (4).

4 $$FC=100-(\%{M}_{c}+ \%Ash+ \%{V}_{m})$$

where $FC$= Fixed carboncontent, $\%{M}_{c}$ = Percentage moisture content, %Ash = Percentage Ash Content, $\%{V}_{m}$ = Percentage Volatile Matter.

### Ultimate (elemental)/calorific value analyses

Ultimate analysis was carried out at the Institute of Agricultural Research and Training, IAR&T, Ibadan, Nigeria, following ASTM standards for tests of biomass materials. A Parr 6200 oxygen bomb calorimeter (Model No: A1290DDEE) was used to analyze the calorific values (higher heating values, HHVs) of the samples using the ASTM D5865-04^30^ standard. An oven-dried 2 g sample from each of the selected biomasses was completely combusted using the ASTM D4239-11^31^ standard. The ultimate analysis was carried out using a LECO-CHN628 Analyser (Model No: 622-000-000), and sulfur content analysis was conducted by using a LECOS-144DR Sulphur determinator (Model No:606-0000-300, SN-477), while the oxygen content was estimated by a positive difference between 100 and the sum of the carbon, C, hydrogen, H, nitrogen, N, sulfur, S and ash contents, AC, using the model in Eq. (5)^32^.

5 $$\% {\text{O }} = { 1}00 \left( {{\text{C}} + {\text{ H }} + {\text{ N }} + {\text{ S }} + {\text{ AC}}} \right)\%$$

### Determination of atomic ratio; C/N, H/C, and O/C

Atomic ratios of C/N, H/C, and O/C were determined by using the model in Eqs. (6), (7), and (8), respectively^33,34^.

6 $$C{:}N \; Ratio= \frac{\%Carbon \; Content}{\%Nitrogen \; Content}$$

7 $$H{:}C \; Ratio= \frac{\%Hydrogen \; Content}{\%Carbon \; Content}$$

8 $$O{:}C \; Ratio= \frac{\%Oxygen \; Content}{\%Carbon \; Content}$$

### Determination of net calorific value

The net calorific value (NCV) of selected biomass were individually determined using Eq. (9)^4^.

9 $$NCV=18.7 \left( 1.0-A C-M C\right)- (2.5M C)$$

where NCV = calorific value (Net), AC = content of ash, MC = content of moisture.

However, leveraging the results of the ultimate analysis, the models (Eqs. 10–12) developed by Yin^35^, Sheng and Azevedo^36^, and Boie^37^ were used to predict the calorific value(s) and CVs of the selected biomass to facilitate easy comparison. HHV is determined in MJ/kg. Individual analysis was carried out in triplicate, and this study reported the average values.

10 $${\text{HHV }} = 0.2949 {\text{C }} + 0.825 {\text{H}}$$

11 $${\text{HHV }} = - {1}.{3675 } + 0.{\text{3137 C }} + 0.{7}00{\text{9 H }} + 0.0{\text{318 O}}$$

12 $${\text{HHV }} = 0.3517{\text{ C }} + 1.1626 {\text{H }} +0.{1}0{\text{47 S }} - 0.{\text{111O}}$$

### Compositional analyses

The gravimetric method was adopted for compositional analyses in accordance with the ASTM E1757-01^38^ standard. Individual analysis was carried out in triplicate, and this study reported the average values. Details are as followed:

### Extractives content determination

A cellulose thimble of Soxhlet extractor was loaded with a dried raw biomass sample of 2.5 g. With the Soxhlet extractor setup (Model No: BST/SXW-6, Bionics Scientific Technologies, India), 150 mL acetone (reagent) was used. This served as a solvent for extraction. With the sample left on the heating mantle for the 4-h run, the stage retention times for boiling and rising were adjusted to 70 °C and 25 min, respectively, with care. After extraction at room temperature and for 5 min, the sample was air-dried. Then, a constant weight of extracted content was attained in a conventional oven at 105 °C. The weight difference between the raw extractive-loaded sample and the control sample (2.5 g) was regarded as the percentage weight, %wt. of the extractives.

### Determination of hemicellulose content

A sample of 1 g extracted dried substrate was poured into a 250 mL Erlenmeyer flask. Then, 150 mL of 500 mol/m^3^ NaOH was introduced. The mixture was boiled together with distilled water for 3½ h. After cooling using vacuum filtration, the sample was filtered and washed until neutral pH was observed. The sample was dried to constant weight at 105 °C in a conventional oven. The hemicellulose content (%wt.) of dry biomass was calculated by obtaining the difference between the sample weight before and after this treatment.

### Determination of lignin content

A 0.3 g sample size of the dried extracted raw substrate was weighed in glass test tubes. Then, 3 mL of 72% H_2_SO_4_ was added. After this, the sample was held at room temperature for 2 h with careful agitation at 30-min intervals to ensure complete hydrolysis. After the initial hydrolysis, 84 mL of distilled ${H}_{2}O$ was added, thus making it slurry. The succeeding step of hydrolysis was initiated using an autoclave (Model No: SSR-2A) for 1 h at 121 °C. Then, the slurry was allowed to cool at room temperature. Hydrolysates were filtered via vacuum filtration with the application of a filtering crucible. Lignin, which is acid insoluble, was established through drying of the residues at 105 °C, and the hydrolyzed samples were incinerated at 575 °C in a muffle furnace to ash. When the UV–VIS spectrophotometer of model no: UH4150AD was used, the absorbance of acid hydrolyzed samples at 320 nm was measured as acid-soluble lignin fraction. Arithmetic addition of acid; insoluble and soluble lignins was regarded as the calculated lignin content.

### Determination of cellulose content

Percentage cellulose content (%wt.) was obtained by difference, with the assumption that extractives, hemicellulose, lignin, cellulose, and ash were the only constituents of the total biomass.

### Determination of cow dung’s bacteria load

This was evaluated in terms of the types and counts of bacteria present in cow dung. A cow dung suspension was prepared using the serial dilution method as contained in the ASTM D5465-16^39^ standard. One gram of cow dung sample was blended in 10 ml sterilized phosphate buffer and shaken extensively in vortexing for 2 min to ensure homogeneity of the sample. Then, the sample was incubated at 37 °C for 30–40 min in an incubator (Model No: CB170) so that the microorganisms could be activated. After incubation, a standard dilution method was used to ensure the dilution of the sample using a sterilized pipette. The prepared phosphate blank contained 9 ml of sterilized phosphate buffer. Having placed the tube in the test tube stand, 1 ml of activated standard solution was transferred aseptically to the test tube.

### Isolation and purification of microorganisms

Various bacterial cultures were then purified using the streak plate method on nutrient agar medium^40^. Using a sterilized inoculating loop, a slightly picked colony from the spread plate was made to drag the loop over the face of another plate in a haphazard motion. The loop was sterilized over the flame, and the plate rotated to 90°, drag the loop over the area initially streaked before. Then, the plate was incubated for 24 h. Isolated colonies and their growths were observed. This procedure was repeated several times until purified colonies were ensured. The purified bacterial culture was kept over nutrient agar slant.

### Microorganism characterization and their identification

Consequently, upon the pure culturing method, all isolated microorganism colonies were investigated under a microscope (Model No: B3070) for colony morphology determination based on varying characteristics, such as size, colour, edges, shape, surface, elevations, and margins. Different stainings (Gram’s staining and endospore staining) were used to identify the cultures^40^.

## Results and discussion

### Characterization of raw lignocellulose biomass

Understanding the chemical compositions of lignocellulose biomass is expedient to maximize the beneficial advantages of such biomass for energy production. Therefore, selected raw biomass, namely, cow dung, mango pulp, and *Chromolaena odorata leaves*, were characterized using the ASTM standard methods, and the results alongside comparisons with those from previous studies are discussed in this section. The proximate analysis included the volatile matter (VM), moisture content (MC), ash content (AC), and fixed carbon (FC); individual heating values were determined in the form of calorific values; ultimate analysis was used to establish the carbon, C, hydrogen, H, nitrogen, N, Sulphur, S and oxygen, O contents; and compositional analysis was employed to investigate the hemicellulose, lignin, and cellulose contents of the selected biomass. The results of bacterial load and counts are also discussed extensively. Table 1 presents the results of proximate, calorific, ultimate, and compositional details of the selected biomass.

**Table 1:** Proximate, calorific, ultimate, and compositional analyses.

Biomass	Cow dung	Mango pulp	*Chromolaena odorata leaves*
**Proximate analysis (%)**			
Volatile matter, (wet basis)	5.02	6.87	7.79
Volatile matter, (dry basis)	60.75	62.78	71.49
Moisture content (wet basis)	85.82	85.77	83.11
Moisture content (dry basis)	10.98	11.35	9.89
Ash content, (wet basis)	1.91	1.14	1.88
Ash content, (dry basis)	5.60	3.85	5.12
Fixed carbon, (wet basis)	7.25	6.22	7.22
Fixed carbon, (dry basis)	22.67	22.02	13.50
VM/FC	0.69	1.10	1.07
**Calorific values (MJ/kg)**			
Calorific value	14.37	13.77	16.16
**Ultimate analysis (%)**			
Carbon, C	43.08	39.98	41.69
Hydrogen, H	7.87	6.74	9.86
Nitrogen, N	1.53	1.34	1.51
Sulfur, S	0.46	0.12	0.25
Oxygen, O	47.06	51.82	46.69
C/N ratio (no unit)	28.16	29.84	27.61
H/C (no unit)	0.19	0.17	0.24
O/C (no unit)	1.09	1.29	1.12
**Compositional analysis (%)**			
Hemicellulose	10.76	7.47	11.37
Lignin	6.33	0.22	0.90
Cellulose	12.03	3.71	5.15
%NDF	41.69	47.90	49.80
%ADF	29.19	40.70	32.78

Values represent the average value for respective analysis.

### Proximate and calorific analyses of cow dung, mango pulp, and *Chromolaena odorata leaves*

Cow dung had the lowest percentage of volatile matter (5.02%), followed by mango pulp, 6.87%, while *Chromolaena odorata leaves* had the highest percentage of 7.79% (as shown in Table 1). Comparatively, percentage volatile matter for cow dung, 66.11% obtained by Tejas^9^ on a dry basis was higher than those recorded in this study for cow dung, 60.75% and mango pulp, 62.78% but lower than that of *Chromolaena odorata leaves*, 71.49%. From Table 1, cow dung had the least percentage of volatile matter both on wet and dry basis, however, it is suggested from the literature that although cow dung has minute percentage of volatile matter, it can produce biogas earlier than any other biomass^41^. This is due to the microorganisms responsible for anaerobic digestion that are readily available in it since cows also feed on green leaves among others^42^. The significant proportion of volatile matter in the selected biomass fuel can be a positive influence for an improved ignition of the dust-air cloud and flame stability^43^. Of all 3 biomasses, cow dung had the highest moisture content, with over four-fifths (85.82%) of its total mass mainly composed of water, which is not unconnected to the fact that the biomass was on a wet basis; thus, this result was expected.

In comparison with other biomasses, a relatively higher percentage moisture content, 85.77%, was recorded for mango pulp, while *Chromolaena odorata leaves* had the lowest moisture content of 83.11% (Table 1). Although bio gasification is accompanied by an anaerobic digestion process, a rated level of moisture content is required to aid microbial activities on the biomass^44^; thus, slurry preparation is essential before incubation. However, the slight differences observed in the values of moisture contents could be attributed to their sources of collections, feeds, nutrients and species. Overall, all three biomasses had adequate and comparable moisture contents, with cow dung having a slightly higher value. Relatively, the VM/FC ratios of the biomass, which range from 0.69 to 1.10, were found to be suitable and imply that a higher reactivity can be achieved since ignition is easier at low temperatures with the aid of volatile matter^45^.

Comparatively, Table 1 shows that cow dung exhibited the highest ash content, 1.91% (on a dry basis), and the lowest volatile matter content, 5.02%. It has been reported that the ash content reduces the fuel quality because ash has an affiliation with fouling^34^; however, high volatile matter ascertains easy ignition^46^; therefore, it would be easier to ignite gas obtained from the digestion of *Chromolaena odorata leaves* with 7.79% volatile matter than either cow dung, 5.02%, or mango pulp, 6.87%. Blending the three biomasses promises to create a means for striking balance in terms of the required volatile matter for effective biogas combustion. On a dry basis, the average percentage ash content of 10.74% obtained for the biomasses investigated in this study is near to those obtained for rice husk (12.75%) by García et al.^47^ and water hyacinth leaves (13.93%) by Jimoh et al*.*^48^ but slightly higher than those obtained for reed canary grass (8.2%) and sorghum (7.2%), as remarked in the study of Lalak et al*.*^43^. Going further, in comparison with the study of Kim et al*.*^49^, where very low percentage ash contents were recorded for wood 2.18% and kenaf 5.45%, these call for critical examination. Although these biomasses are lignocellulosic, the differences observed in the results could be attributed to the type, nature, and sources of the biomass; therefore, adequate attention should be given to the nature and sources of biomass collection to be implemented for anaerobic digestion to ensure methane-rich biogas. Cow dung and mango pulp have been found in the literature and established in this study to be suitable biomasses for biogas production. When the duo is digested with *Chromolaena odorata leaves*, it becomes viable for yielding more methane than being digested individually. It could also be deduced from Table 1 that there exists a directly proportional relationship between ash content and fixed carbon across the selected biomass, i.e., an increase in ash content brings about an increase in fixed carbon and vice versa. In contrast, an inversely proportional relationship exists between volatile matter and moisture content, MC of the biomass, such that the higher the volatile matter, VM, the lower the moisture content and vice versa. The results further revealed a negligible difference between the fixed carbon contents of cow dung (7.25%) and *Chromolaena odorata leaves* (7.22%). This suggests that since cows also feed on *Chromolaena odorata leaves* (either completely or as a supplement) or any other green leaves for diet, the dung had similar characteristics as that of the raw *Chromolaena odorata leaves*^50,51^. Generally, the efficacy of the anaerobic digestion process has a significant relationship with the types and characteristics of biomass. Furthermore, characteristics of the biomass were discovered to have a great impact on degradation and retention times^52^. The calorific value is the quantity of energy that is generated per unit of mass per unit volume of the biogas fuel when it is completely burnt in the presence of oxygen ^53^. Additionally, there were technical agreements between the contents of volatile matter and calorific values obtained for cow dung, 14.37 MJ/kg, mango pulp 13.77 MJ/kg, and *Chromolaena odorata leaves*, 16.16 MJ/kg, respectively. Evidently, from Table 1, it could be said that volatile matter and calorific values are proportionate. All 3 biomasses had attractive and significant calorific values, but *Chromolaena odorata leaves* had the highest percentage of volatile matter, which subsequently suggested why they had the highest calorific value. Although Boie’s model is the best and well suited for predicting the calorific values of typical biomass, this study used the ultimate analysis as input variables in three model equations (i.e., Yin^35^; Sheng and Azevedo^36^; Boie^37^) to predict the calorific values of the investigated biomass. In comparison with the experimental results of this study, all the predicted calorific values had significant increments, such that percentage deviations of 26.00–33.59%, 24.42–33.32%, and 17.71–33.42% were recorded using the models of Yin^35^, Sheng and Azevedo^36^, and Boie^37^, respectively. This suggests that further studies are still required for the prediction of biomass calorific values^54^; however, the use of calorimetry for the determination of calorific values remains important.

**Figure 2:**
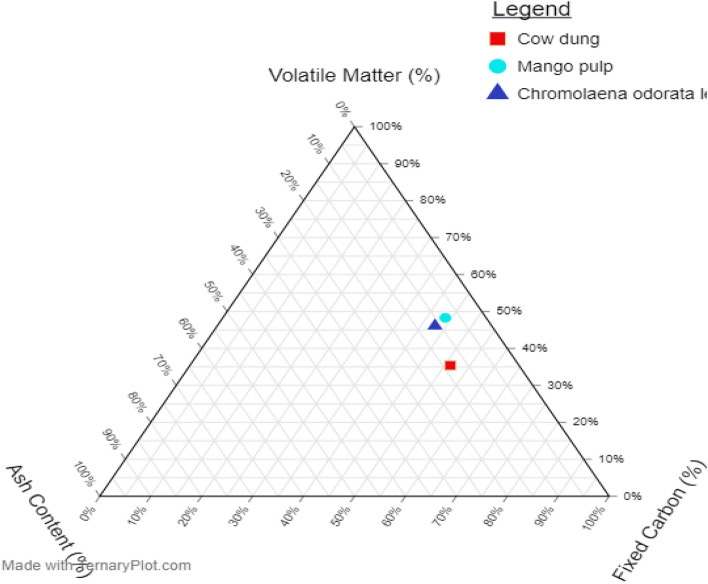
Ternary plot of volatile matter (%), ash content (%), and fixed carbon (%) on a wet basis.

A ternary plot is a triangular coordinate system having the edges of the triangle as the axes where each edge corresponds to a composition of the system. Figure 2 presents a ternary plot that was used to visualize and understand the overall proximate compositional variations of the investigated biomass. The mean proximate compositions of the sampled biomass were plotted using the principles of Singh et al*.*^53^ and Vassilev et al*.*^55^. Specifically, three key data were plotted: the volatile matter, VM, ash content, and fixed carbon, FC, all in percentages^55^. Critical examination of the plot showed proximity among the sampled biomass, which is an indication of the similarities in the chemical components of the biomass^53^. Thus, these results suggest their suitability for energy production, especially when blended.

### Ultimate analysis of biomass

**Figure 3:**
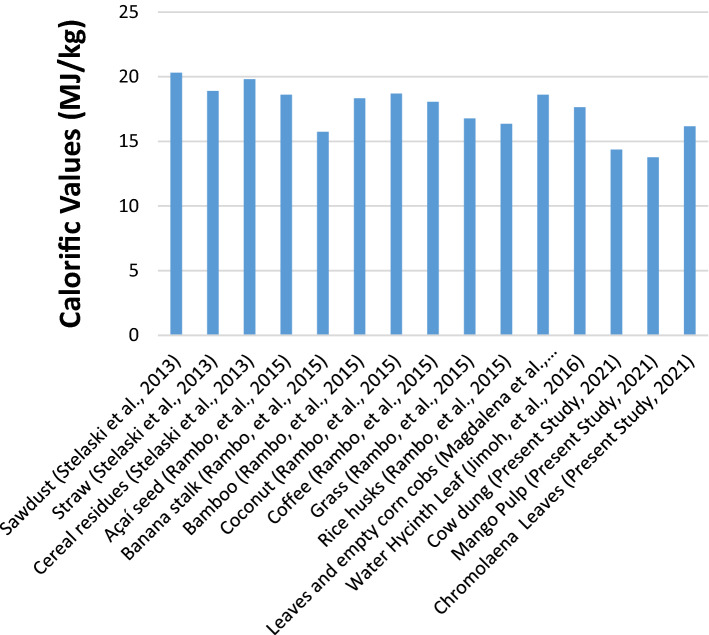
Calorific values for previous and present studies.

One of the fast-growing alternative renewable energy forms that could serve as a replacement for fossil fuels is biomass energy. The suitability and quality of biogas to be produced by any selected biomass could be informed by ultimate analysis; hence, these factors necessitated the elemental, CHNSO, and investigative contents shown in Table 1. Carbon (39.98–43.08%), hydrogen (6.74–9.86%), nitrogen (1.34–1.53%), Sulfur (0.12–0.46%), and oxygen (46.69–51.82%) of cow dung, mango pulp, and *Chromolaena odorata leaves* are similar to those obtained for lignocellulose biomass investigated in past studies (Okolie et al.^5^; Kobra et al.^13^; Wannapokin et al.^14^; Dahunsi et al.^34^; Adegun and Yaru^56^: Fang et al*.*^57^). It is clear that all the biomass has low carbon contents and high oxygen contents, which are consistent with those reported for grasses and manure by Harpreet^58^ but inconsistent compared to coal by the same study. The results also indicated that the higher the oxygen concentration of typical biomass was, the lower the carbon content, which is observable in Table 1. Nitrogen and sulfur contents are reported not to be important in biofuel production because they tend to release harmful and toxic gases^48^. Therefore, negligible sulfur contents obtained for the selected biomass suggest their suitability for biogas production with a minute possibility of releasing voluminous toxic gases, which could negatively affect humans and the environment. Percentage contents of sulfur (0.46, 0.12, and 0.25) % and nitrogen (1.53, 1.34, and 1.51) % for cow dung, mango pulp, and *Chromolaena odorata leaves,* respectively, are considered acceptable because they imply low concentrations of oxides of sulfur and nitrogen present in biogas obtained as a result of their digestion^53^. Therefore, during biogas combustion, the possibility of releasing toxic gases that would otherwise cause undesirable environmental impacts is infinitesimal. These results match those obtained for similar lignocellulose biomass considered in the research conducted by Singh et al.^53^. Furthermore, the range of calorific values (13.77–16.16 MJ/kg) is indeed interesting and is found to be in accordance with the proximate and ultimate analyses that depict favourable levels of fixed carbon contents, i.e., above 13% and ash contents of less than 2%, respectively; with these results, the carbon contents promise to positively contribute to increasing the calorific values^45^. From Fig. 3, the calorific values of the selected biomass were all consistent with those obtained in previously studied lignocellulose biomass Jimoh et al*.*^48^; Rambo et al*.*^59^; Magdalena et al*.*^60^; Stelaski, et al*.*^61^. These results suggest the characteristic similarities of lignocellulosic biomass reported in the literature; however, slight variations observed in the calorific values presented in Fig. 3 could be ascribed to the locality of the biomass, climatic and environmental states, discrepancies in determination processes, nutrition, and chemical structure of the types of biomass investigated^48,49,62^. The predicted calorific values (using Eqs. 10–12, developed by Yin^35^, Sheng and Azevedo^36^, and Boie^37^, respectively) from those experimentally obtained in this study. This could be a result of inadequate data used for modeling on the part of the authors who developed the models because the greater the data point, the greater the coefficient of correlation (R-square value) tends towards unity and consequently enhancing the accuracy of the model.

### Carbon–nitrogen (C/N) ratio

The estimated carbon/nitrogen ratios of selected biomass ranged between 27.61 and 29.84, with cow dung and *Chromolaena odorata leaves* having nearly the same value of approximately 28 each, although with a slight difference of 0.24, while mango pulp had the highest ratio of 29.84 (see Tables 1 and 2). Previous studies have shown that anaerobic bacteria source their foods from carbon and nitrogen such that carbon is needed for energy, while a combination of carbon and nitrogen is used for building new cell structures^63^. The process of anaerobic digestion is inclined to be the proportion of carbon/nitrogen present because it is an indication of the nutrient level of the biomass^64^. Moreover, a high C/N ratio tends to cause insufficient nitrogen for maintaining biomass(es) cells and consequently brings about an ammonia nitrogen supply in the digester^64^. Additionally, a high C/N ratio is an indication of rapid consumption of nitrogen by methanogens, which results in lower gas production^65^. In contrast, a low C/N ratio can lead to possible ammonium inhibition of microorganism activities in the digester, resulting in an optimum pH value to exceed 8.5, which is toxic to methanogenic bacteria^64,65^. Thus, to strike a balance between the two levels (high/low) of C/N ratios, a combination of biomass with considerably low and high ratios of C/N is proposed, such as organic waste blended with sewage or animal manure; thus, the biomass investigated in this study can form a good blend for biogas production. The advantages of this approach include not only having an optimum operational C/N ratio but also having a higher methane content yield when codigested compared to sole digestion^49^. Practically, several previous studies had an optimum biomass C/N ratio for anaerobic digestion, ranging between an average value of 20 and 35^63,64^. All the respective C/N ratios obtained in this study, that is, cow (28.16), mango pulp (29.84) *Chromolaena odorata leaves* (27.61), and the overall average (28.4) recorded, fell within the optimum range found in the literature. This formed the basis upon which the biomass could be considered an excellent anaerobic digestive for biogas production^63,64^. Although the C/N ratios recorded for this present study fall within the optimum range, they varied from those reported for rice husk (66.17) and walnut shells by Hongtao et al*.*^66^ and wood pellet (84.33) by Kim et al*.*^49^. The variations may be due to different feedstocks given to the cows, soil compositions, and climatic conditions^9,60^.

**Table 2:** The ultimate analysis compared to some other selected lignocellulose biomass.

Biomass	C	H	N	S	O	C/N	H/C	O/C	References
Cow dung	43.08	7.87	1.53	0.46	47.06	28.16	0.18	1.09	Present study
Mango Pulp	39.98	6.74	1.34	0.12	51.82	29.84	0.17	1.30	Present study
Chromolaena odorata	41.69	9.86	1.51	0.25	46.69	27.61	0.24	1.12	Present study
Cow dung	38.95	5.05	1.573	< 2.00	34.36	24.76	0.13	0.88	Tejas^9^
Hard coal	75.70	4.30	1.20	1.20	5.90	63.08	0.06	0.08	Lalak^43^
Sorghum	45.60	5.70	0.90	0.00	32.70	50.67	0.13	0.72	Lalak^43^
Reed canary grass	44.90	5.80	0.90	0.10	31.90	49.89	0.13	0.71	Lalak^43^
Miscanthus	48.40	6.00	0.40	0.00	34.20	121.00	0.12	0.71	Lalak^43^
Brome grass	46.20	6.00	0.60	0.00	34.60	77.00	0.13	0.75	Lalak^43^
Wheat straw pellet	49.40	5.60	0.60	0.10	35.70	82.33	0.11	0.72	Lalak^43^
Pellet bamar	46.70	5.9	0.60	0.00	33.50	77.83	0.13	0.72	Lalak^43^
Wood pellet	48.07	6.62	0.57	0.06	44.68	84.33	0.14	0.93	Kim^49^
Kenaf	46.71	6.71	1.21	0.05	45.32	38.60	0.14	0.97	Kim^49^
Impereta cylindrical	50.03	5.92	1.14	NR	42.89	43.89	0.12	0.86	Singh et al.^53^
Eragrostis airoides	41.02	6.72	1.13	NR	51.11	36.30	0.16	1.25	Singh et al.^53^
Typha angustifolia	52.89	5.84	1.21	NR	40.04	43.71	0.11	0.76	Singh et al.^53^
Arundinella khasiana	41.26	5.38	1.25	NR	52.09	33.01	0.13	1.26	Singh et al.^53^
Echinochloa stagnina	44.98	5.66	1.85	NR	47.49	24.31	0.13	1.06	Singh et al.^53^
Açaí seed	47.60	6.4	0.78	NR	45.12	61.03	0.13	0.95	Rambo et al.^59^
Banana stems	39.00	5.44	0.82	NR	54.84	47.56	0.14	1.41	Rambo et al.^59^
Banana stalk	37.95	4.73	1.46	NR	55.85	25.99	0.12	1.47	Rambo et al.^59^
Bamboo	44.60	5.55	0.91	NR	48.93	49.01	0.12	1.10	Rambo et al.^59^
Coconut	47.40	5.41	0.55	NR	46.64	86.18	0.11	0.98	Rambo et al.^59^
Coffee	43.34	5.5	2.25	NR	48.86	19.26	0.13	1.13	Rambo et al.^59^
Sawdust	50.30	6.08	0.15	NR	43.43	335.33	0.12	0.86	Rambo et al.^59^
Grass	42.00	5.21	2.03	NR	50.95	20.69	0.12	1.21	Rambo et al.^59^
Rice husks	35.86	5.21	0.28	NR	59.46	128.07	0.15	1.66	Rambo et al.^59^
Soy peel	45.04	6.7	2.90	NR	45.35	15.53	0.15	1.01	Rambo et al.^59^
Bagasse	51.71	5.32	0.33	NR	42.64	156.70	0.10	0.82	Mosiori et al.^62^
Paddy straw	48.75	5.98	1.99	NR	43.28	24.50	0.12	0.89	Mosiori et al.^62^
Wood stem	50.52	5.81	0.23	NR	43.44	219.65	0.12	0.86	Mosiori et al.^62^
Eucalyptus grandis	45.50	5.55	0.17	NR	48.39	267.65	0.12	1.06	Mosiori et al.^62^
Walnut shells	52.62	5.67	0.34	0.11	41.25	154.76	0.11	0.78	Hongtao et al.^66^
Rice husks	50.95	7.00	0.77	0.24	41.04	66.17	0.14	0.81	Hongtao et al.^66^
Palm kernel shell	47.88	5.15	0.94	0.10	42.69	50.94	0.11	0.89	Onochie et al.^67^
Palm fibre	42.20	5.21	2.22	0.14	42.34	19.01	0.12	1.00	Onochie et al.^67^
Empty fruit bunch	43.89	5.33	0.51	0.10	54.32	86.06	0.12	1.24	Onochie et al.^67^
Straw pellet	49.52	5.72	0.77	0.13	37.54	64.31	0.12	0.76	Işık-Gulsac et al.^68^
Softwood pellet	54.30	5.8	0.002	0.03	39.30	27,150.00	0.11	0.72	Işık-Gulsac et al.^68^
Milled sunflower seeds	48.86	5.8	4.78	0.57	31.87	10.22	0.12	0.65	Işık-Gulsac et al.^68^

*NR* not reported.

### Determination of energy densities using H/C and O/C atomic ratios

**Figure 4:**
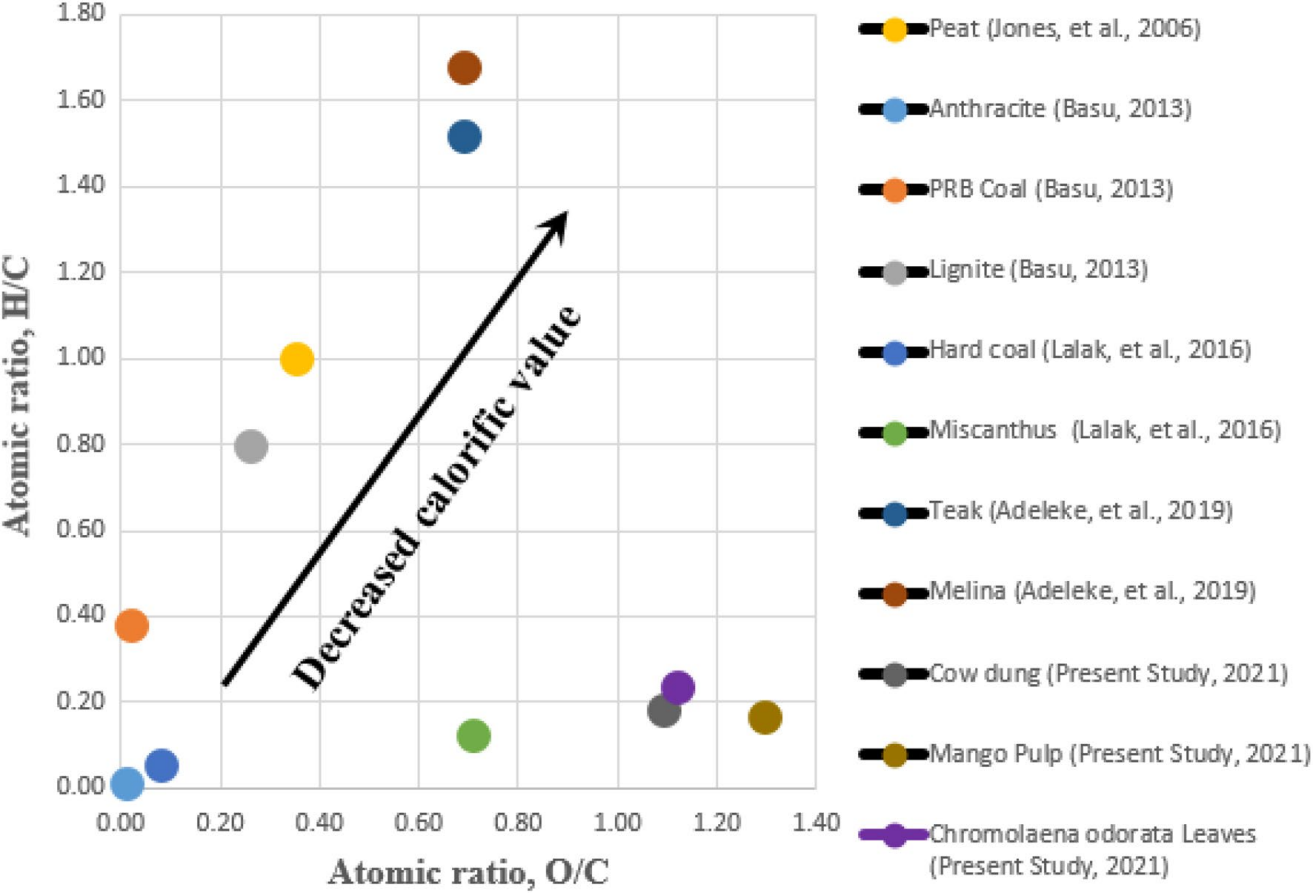
Van Krevelen diagram showing atomic ratios of H:C against O:C for past and present studies.

Naturally, asides from the proximate and ultimate analyses of biomass, lignocellulosic biomass also differ from one another based on their compositional formulations, which is a function of fuel efficacy, i.e., ability to produce adequate energy in forms of heat and electricity^33^). The classification of atomic ratios, involving hydrogen, oxygen, and carbon, such as H:C and O:C atomic ratio diagrams, is a useful approach that can be used to understand the fuel calorific value^54,69^. A plot of the Van Krevelen diagram shown in Fig. 4 was plotted with respect to atomic ratios of hydrogen to carbon, H/C against oxygen to carbon, and O/C, and the correlations between them were used to locate the energy density (in terms of individual position relative to one another) of eleven (including those of this study) selected biomass samples in the Van Krevelen diagram presented in Fig. 3. It has been reported that there is a directly proportional relationship between atomic ratios and the energy content of biomass fuel ^70^, which implies that the energy density of fuel biomass has a direct correlation (inverse proportional relationship) with atomic ratios, i.e., H/C and O/C. Compared to atomic ratios of cow dung 0.18 H/C and 1.09 O/C, mango pulp 0.17 H/C and 1.30 O/C, the atomic ratios H/C and O/C of *Chromolaena odorata* leaves are 0.24 and 1.12, respectively. Therefore, *Chromolaena odorata* leaves had the highest value of H/C, 0.24, and mango pulp with the least H/C value of 0.17 but with the highest value of O/C 1.30, while cow dung recorded the least O/C value of 1.09 compared to others. These ratios were observed to be higher than those of other types of fuels, anthracite, 0.01 H/C, 0.01 O/C^70^, hard coal 0.06 H/C, 0.08 O/C^43^, and miscanthus 0.12 H/C, 0.71 O/C^43^ (Fig. 3). Comparatively, high hydrogen/carbon and oxygen/carbon atomic ratios were the factors that lowered the heating values of cow dung, mango pulp, and *Chromolaena odorata* leaves, respectively. However, it caused an increase in the heating values when compared to PRB coal, lignite, peat, teak, and melina^54,70,71^, where the atomic ratios for this study were observed to be lower in decreasing order of magnitude of the calorific values (Fig. 3). This implies that, compared to those of this study, higher atomic ratios (H:C and O:C) were recorded for other biomasses. Digestion feedstock with relatively low O:C ratios have more energy densities with higher calorific values; thus, comparatively, greater chemical energy is obtainable in C–C bonds than in C-O bonds^53^. The calorific values obtained in this study are traceable to the percentage of fixed carbon as a result of lower O:C and H:C atomic ratios. Table 2 reveals that all ratios obtained for this study fall within those compiled and adapted from the literature. Thus, the biomass investigated can be adopted for biogas production or processed into the desired renewable energy form.

### Compositional analysis

Typical lignocellulose biomass, such as those investigated in this study, primarily comprises cellulose, hemicellulose, and lignin^51^. Although they have associated virtues with each other, their performances under anaerobic digestion are different. Accurate compositional analysis of lignocellulose biomass gives room for evaluating the conversion yields as well as process economics, especially in biogas conversion processes^11^. The percentage hemicellulose, lignin and cellulose contents of the biomass were cow dung (10.76, 6.33, 12.03) %, mango pulp (7.47, 0.22, 3.71) % and *Chromolaena odorata leaves* (11.37, 0.90, 5.15) %, respectively (Table 1). It has been reported that the considerable cellulose and lignin contents should be relatively small if digestion is to be aided because those contents are not easily bioconvertible in anaerobic environments as a result of their rigid structure^72^. This hinders the anaerobic digestion process and consequent reduction in the rate of biogas generation; therefore, the higher the lignin content becomes, the lower the corresponding biogas yield^72,73^. However, to obtain energy from combustion, it is stated that a considerably larger amount of lignin is preferable, as a higher calorific value of biomass has a strong positive correlation with lignin content. From Table 3, generally, the content of lignins present in nonwoody biomass was discovered to be lower but higher in woody biomass. Cow dung had the highest lignin and cellulose values compared to other biomasses. Additionally, it is characterized by a higher calorific value because a higher calorific value is associated with higher lignin and extractives^59^, while similar trends were observed for *Chromolaena odorata* leaves followed by mango pulp. The considerable amounts of lignin, hemicellulose and cellulose contents in the selected biomass are indications that when harnessed adequately, they can produce useful energy through anaerobic digestion. Similarly, the slight difference observed between lignin concentrations of mango pulp and *Chromolaena odorata* leaves could be attributed to the nutrients of the hosting trees. All three biomasses characterized had significant cellulose contents, with mango pulp having the lowest value of 3.71%, while that of cow dung topped the list with a value (12.03%) more than twice that of *Chromolaena odorata leaves* (5.15%) and more than thrice that of mango pulp (3.71%). Variations due to the types and nature of biomass were observed between the results of compositional analysis recorded for this study and those found in the literature (Table 3).

**Table 3:** Comparison of compositional analysis of previous and present studies.

Biomass	Cellulose (wt.%)	Hemicellulose (wt%)	Lignin (wt%)	References
Wheat straw	35.0–49.09	23.0–30.0	12.0–16.0	Harpreet^58^
Miscanthus	38.80	36.30	11.50	Harpreet^58^
Willow	49.05	31.50	14.73	Harpreet^58^
Rice husks	28.7–35.6	12.0–29.3	15.4–20.0	Hongtao et al*.*^66^
Wall nut	45.1	24.2	23.1	Hongtao et al*.*^66^
Sugarcane bagasse	25.0–45.0	28.0–32.0	15.0–25.0	Bajpai^74^
Sorghum straw	32.0–35.0	24.0–27.0	15.0–21.0	Bajpai^74^
Barley straw	36.0–43.0	24.0–33.0	6.3–9.8	Bajpai^74^
Grasses	25.0–40.0	25.0–50.0	10.0–30.0	Bajpai^74^
Switch grass	35.0–40.0	25.0–30.0	15.0–20.0	Isikgor et al*.*^75^
Corn cob	33.7–41.2	31.9–36.0	6.1–15.9	Lin et al*.*^76^
Corn stalk	35.0–39.6	16.8–35.0	7.0–18.4	Lin et al*.*^76^
Rice straw	19.8 – 34.7	23.0–43.9	17.0–29.3	Lin et al*.*^76^
Hardwood (poplar)	50.8–53.3	26.2–28.7	15.5–16.3	Jørgensen et al*.*^77^
Softwood (pine)	45.0–50.0	25.0–35.0	25.0–35.0	Jørgensen et al.^77^
Cow dung	12.03	10.47	6.33	Present study
Mango pulp	3.71	7.47	0.22	Present study
Chromolaena odorata	5.15	11.37	0.90	Present study

### Results of microbial load

Furthermore, to ensure the suitability of any biomass for anaerobic digestion, specifically manure, this study characterized cow dung for bacterial load and count, which was determined as colony-forming units per gram (cfu/g). An important factor that can inform the quality of any manure is the total viable count because changes in microbial varieties may result in changes in dung functionality in the digester throughout the digestion process^78^. Thus, the results obtained for this characterization revealed that cow dung has total bacterial counts of 5.78 × 10^8^ and 3.93 × 10^5^ on wet and dry bases, respectively. It was also clear from the results obtained that the fresh cow-dung sample was enriched in microbial colonies, evidently from the various species found, such as *Escherichia coli, Staphylococcus aureus, Bacillus cereus, Pseudomonas aureginosa, Proteus morganii, and Micrococcus* spp. The microbial contents of cow dung may explain its bioefficacy, thus justifying its usage for biogas production^79^. Additionally, manure quality and suitability as feedstock depend on microbial presence, and the digestate of cow dung when codigested with other lignocellulose biomass could serve as a potential fertilizer.

## Conclusions and recommendation

The suitability of the huge biomass available (especially Cow dung, Mango pulp, and *Chromolaena odorata* leaves) for energy conversion has been established, since the advantages of biomass in diversity can be used to augment the present global energy mix. This was established through the results of various characterizations, such as proximate and ultimate calorific values and compositional and microbiological analyses, which all met the requirements for suitable feedstock(s) available in the literature. The energy contents in the form of calorific values obtained for the characterized biomass, cow dung, mango pulp, and *Chromolaena odorata* leaves were 14.37, 13.77, and 16.16 MJ/kg, respectively. The ultimate analysis of cow dung, mango pulp and Chromolaena odorata recorded carbon (43.08, 39.98, 41.69%); hydrogen (7.87, 6.74, 9.86%); nitrogen (1.53, 1.34, 1.51%); sulphur (0.46, 0.12, 0.25%) and oxygen (47.06, 51.82, 46.69%), respectively. Compositional analysis of the biomass gave percentages in the range of 7.47–11.37 for hemicellulose, 0.22–6.33 for lignin, and 3.71–12.03 for cellulose, while the microbial analysis of cow dung gave total bacteria counts of 5.78 × 10^8^ and 3.93 × 10^5^ cfu/g on wet and dry bases, respectively, which implied that it was rich in microbial colonies, evidently from the various species found, such as *Escherichia coli, Staphylococcus aureus, Bacillus cereus, Pseudomonas aureginosa, Proteus morganii, and Micrococcus *spp. These results conform with the proximate and ultimate analyses that depict favourable levels of fixed carbon contents above 13% and ash contents of less than 2%, respectively; with these, the carbon contents promise to positively contribute to increasing the calorific values. It is therefore recommended that cow dung, mango pulp, and *Chromolaena odorata* leaves be developed as sources of energy that can be applied either for domestic or industrial purposes.

## Data Availability

Upon request, data implemented in this study will be made available.
